# Effect of the SARS-CoV-2 pandemic on metabolic control in patients with type 2 diabetes: a 5-year cohort follow-up managed by a dynamic multidisciplinary team in Northeastern Mexico

**DOI:** 10.1186/s13098-024-01318-8

**Published:** 2024-04-25

**Authors:** Devany Paola Morales-Rodriguez, Arnulfo González-Cantú, Arnulfo Garza-Silva, Andrea Rivera-Cavazos, Iván Francisco Fernández-Chau, Andrea Belinda Cepeda-Medina, Miguel Angel Sanz-Sánchez, Gerardo Francisco del Rio-Parra, María Angelina Torres-Fuentes, Miguel Assael Rodriguez-Puente, Maria Elena Romero-Ibarguengoitia

**Affiliations:** 1 Reseach Deparment, Hospital Clinica Nova de Monterrey, San Nicolas de los Garza, Nuevo Leon Mexico; 2 Medical School, Vicerrectoria de Ciencias de la Salud, Universidad de Monterrey, San Pedro Garza Garcia, Nuevo Leon Mexico

**Keywords:** Type 2 diabetes, Metabolic control, COVID-19

## Abstract

**Background:**

The SARS-CoV-2 pandemic brought a radical shift in the healthcare system and suboptimal care for vulnerable patients, such as those with Type 2 Diabetes Mellitus (T2D). Therefore, we compared metabolic control and macro/microvascular complications of patients with T2D before and throughout the three-year SARS-CoV-2 pandemic.

**Research design and methods:**

A retrospective observational cohort of subjects with T2D studied from 2018 to 2022 in Northern Mexico was treated by a dynamic multidisciplinary team. Levels of Glycated hemoglobin (HbA1c), fasting serum glucose (FG), LDL-Cholesterol (LDL-C), blood pressure (BP), albuminuria, triglycerides, Body Mass Index (BMI), and FIB-4 score, micro and macrovascular complications were evaluated.

**Results:**

A total of 999 patients were studied, 51.7% males with a mean (SD) age of 60.1 (12.7) years. Adequate glycemic control based on HbA1c increased by 15.2% and 42.3% in FSG (p < 0.001) between the beginning 2018 and the end of 2022. LDL-C control decreased by 5.1% between 2018 and 2022 (p < 0.001). Systolic BP control decreased by 2.6% (p < 0.001), whereas diastolic BP control increased by 1.8% (p = 0.01) between 2018 and 2022. Albuminuria control increased by 8.5% (p = 0.002). When comparing the Area Under the Curve (AUC) of metabolic parameters between patients who developed SARS-CoV-2 vs. those who did not, AUC was statistically higher in those who developed SARS-CoV-2 (p < 0.05). Diabetic neuropathy was the most prevalent microvascular complication (n = 35; 3.6%); ischemic heart disease was the most frequent macrovascular complication (n = 11;1.1%).

**Conclusions:**

A multidisciplinary dynamic team that adapts to the pandemic SARS-CoV-2 maintains and increases metabolic control in subjects with type 2 diabetes in Mexico. This represents a low percentage of chronic complications. The AUC of metabolic parameters of subjects with SARS-CoV-2 infection is higher, reflecting more variability in metabolic control.

**Graphical Abstract:**

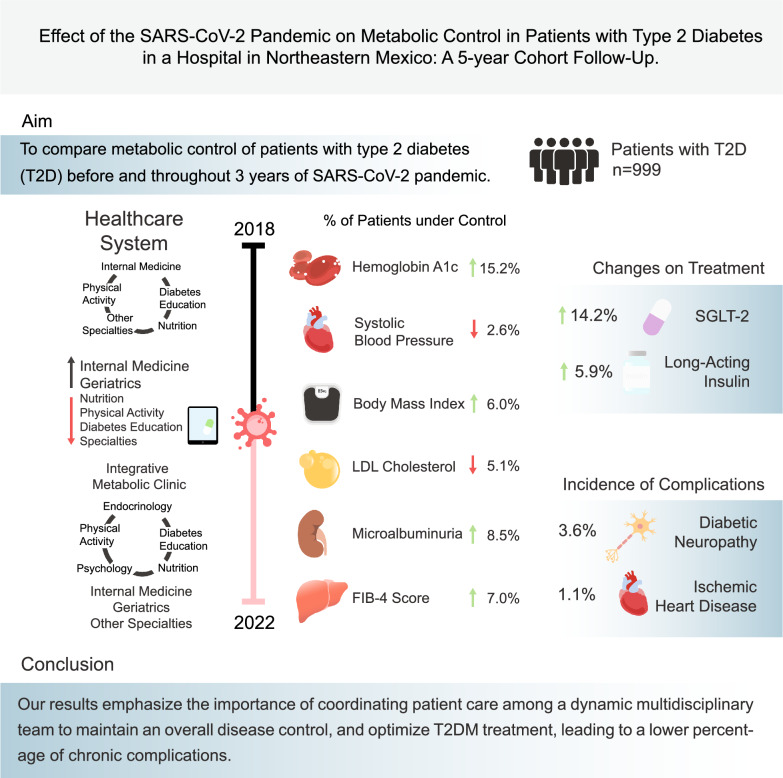

## Effect of the SARS-CoV-2 pandemic on metabolic control in patients with type 2 diabetes: a 5-year cohort follow-up in a hospital in Northeastern Mexico

Type two diabetes (T2D) is a highly prevalent disease worldwide, associated with acute and chronic complications and a high risk of mortality [1, 2]. In 2020, due to the SARS-CoV-2 pandemic, patients with T2D were subjected to a lockdown, leading to changes in lifestyle and physical activity due to the closure of non-essential public places [3]. Moreover, this isolation posed a challenge in traditionally delivering health services, leaving a portion of the population without primary care access. This situation triggered a revolution in healthcare delivery, leading to the implementation of new forms of providing medical attention, such as telemedicine [4].

A 2022 meta-analysis involving nearly 4,000 patients across several countries highlighted worsened fasting serum glucose levels in T2D patients during lockdowns [5]. Consequently, it is of interest to study the effect of the SARS-CoV-2 pandemic on the metabolic control of T2D patients in other populations, such as the Latino population. Limited long-term research regarding multidisciplinary diabetes management exists in Mexico. A previous study conducted in Mexico City with 133,662 patients showed that individuals with type 2 diabetes had generally poor disease control (HbA1c average of 8.9%) [6]. The long-term effect of the pandemic on metabolic control and micro and macrovascular complications associated with T2D in this population is poorly known, which motivates this current study. This research aimed to compare the metabolic control [(mean, proportion of patients at goal and area under the curve (AUC)] and liver fibrosis using FIB-4 score two years before and during three years of the SARS-CoV-2 pandemic in patients with T2D. Secondarily, the study also explored consultation trends, medication usage, micro and macrovascular complications, and the incidence of acute complications during this studied period.

## Research design and methods

This was a retrospective observational cohort study conducted at a hospital in Northern Mexico in subjects with T2D diabetes who had available information in the medical record during the follow-up period from 2018 to 2022. This study was approved through the Institutional Review Board (IRB) of Universidad de Monterrey (10,112,022-CN-MI-CI). Additionally, this study followed STROBE guidelines (Strengthening the Reporting of Observational Studies in Epidemiology) and the Declaration of Helsinki [7].

### Procedure

Information from 2018 to 2022 was extracted from the electronic medical records. The inclusion criteria were patients with type 2 diabetes, aged ≥ 18 years, who had at least one measurement in each year from 2018 to 2022 of glycated hemoglobin (HbA1c) [control considered as < 7% in < 65 years, < 7.5% for adults > 65 years and < 8% in adults > 65 with multiple comorbidities [8, 9]^]^. Additionally, we evaluated the mean, standard deviation (SD), the proportion of control, and area under the curve (AUC) of the following variables: Fasting Glucose (FG) [control considered between 80 and 130 mg/dL] [8], Low-Density Lipoprotein Cholesterol (LDL-C) [control when ≤ 100 mg/dL] [10], High-Density Lipoprotein Cholesterol (HDL-C) [control < 50 mg/dl for women and < 40 mg/dL for men [10]], Triglycerides [control considered when < 150 mg/dL] [10], albuminuria [control < 30 mg/g] [11], with a Roche Cobas 6000 c module 501 (USA); blood pressure [control when < 130/80 mmHg] [10], Body Mass Index (BMI) [control considered between 18.5 and 24.9 kg/m^2^] [12] and Fibrosis 4 Index (FIB-4) for liver fibrosis according to ADA 2024 guidelines [< 1.30 as F0–F1, > 1.30 but < 2.67 as F2 and > 2.67 as F3–F4] [1]. Additionally, the type of consultations (in-person and telephonic), annual attendance in specialty consultations, medication usage, and complications (acute, microvascular, macrovascular) were also evaluated. Other variables that were included were age, gender, personal medical history of systemic arterial hypertension, dyslipidemia, and SARS-CoV-2 infection. A careful and systematic review of the clinical record was performed to avoid bias during data collection. The clinical parameters such as weight, height, and blood pressure were obtained from the medical record when the patient was assisted to an “in-person” medical consultation and had no acute infection that could have biased the results.

A convenience sampling method was employed, including all patients who met the inclusion criteria.

### Hospital Clinica Nova care model

Hospital Clinica Nova is a private hospital located in Northern Mexico. During 2018 and 2019, diabetes management involved in-person consultations with a multidisciplinary team, including the internal medicine department, physical activation specialists, nutritionists, diabetes educators, and specialties, if required, such as ophthalmologists, endocrinologists, cardiologists, nephrologists, etc. In 2019, the Geriatrics department started treating patients older than 70 years, and in 2021, those older than 65. With the onset of the SARS-CoV-2 pandemic, in-person consultations were limited. Over the first two years of the pandemic, telephone consultations were mainly implemented for internal medicine, geriatrics, and endocrinology, with a 70% reduction in other specialties such as nutrition, physical activation, and diabetes education. In-person consultations for nephrology, cardiology, neurology, and ophthalmology were restricted to complex cases. Face-to-face consultations were gradually reintroduced from the third year of the contingency. In September 2022, Hospital Clinica Nova launched its Metabolic Clinic, providing comprehensive care for diabetes, dyslipidemias, and associated complications through a multidisciplinary team comprising internal medicine, endocrinology, obesity clinic, nutrition, physical activity, psychology, diabetes education, and other specialties if required.

### Statistical analysis

The distribution of variables was explored through Kolmogorov–Smirnov and Shapiro–Wilk tests, as well as frequency histograms. Numerical data was normalized through logarithmic transformation. Data was reported as mean, standard deviation (SD), and frequencies and percentages for descriptive statistics. Cochran’s Q test was employed to determine significant differences between categorical data over time, the Chi-square test for independent categorical data, and repeated measures ANOVA for comparing quantitative variables over time. Mann–Whitney U test was computed to calculate the Area Under the Curve (AUC) difference between subjects with SARS-CoV2 infection vs. those that did not have it. Missing data was handled by complete case analysis. Sample size was calculated using G Power vs. 3.1 software (Germany) based on ANOVA of repeated measures formula. Calculations included an effect size f of 0.25, an alpha of 5%, a power of 95%, 10 groups and 10 measurements, and a correlation among repeated measures of 0.5. The minimal sample size required was 220 subjects. Data missing completely at random was analyzed through complete case analysis. Data analysis was conducted using SPSS25 (SPSS Inc. Chicago, IL, USA), with a significance level set at a p-value < 0.05.

## Results

A total of 999 patients were studied, 516 (51.7%) males, with a mean (SD) age of 60.1 (12.7) years. Regarding patients’ chronic comorbidities, 899 (89.9%) patients had dyslipidemia, 684 (68.4%) had hypertension, and 640 (64%) were reported with obesity. Between 2020 and 2022, 396 (39.6%) patients were diagnosed with SARS-CoV-2; 81 (8.1%) in 2020, 174 (17.4%) in 2021 and 197 (19.7%) in 2022.

The mean (SD) of FG had its highest peak in 2018 [163.8 (1.3) mg/dL] and its minimum peak in 2021 [124.8 (1.3) mg/dL], p < 0.001. There were no significant changes in mean HbA1c, cholesterol, triglycerides, BMI, and blood pressure values during the 5-year study (Table 1)**.**

**Table 1:** Average control values in patients from 2018 to 2022

Laboratory Parameter Mean (SD)	Beginning 2018	End of 2018	Beginning 2019	End of 2019	Beginning 2020	End of 2020	Beginning 2021	End of 2021	Beginning 2022	End of 2022	p-value
Fasting Serum Glucose (mg/dL)	163.8 (1.3)^a^ n = 796	159.8 (1.3)^a^ n = 775	124.9 (1.4)^b^ n = 869	126.5 (1.4)^b^ n = 890	127.9 (1.4)^c^ n = 768	126.0 (1.4)^c^ n = 843	128.5 (1.4)^c^ n = 833	124.8 (1.3)^c^ n = 479	132.3 (1.4)^b^ n = 747	130.2 (1.4)^b^ n = 726	< 0.001
Glycated Hemoglobin (%)	7.3 (1.2) n = 888	7.4 (1.2) n = 582	7.2 (1.2) n = 320	7.2 (1.2) n = 956	7.3 (1.2) n = 406	7.1 (1.2) n = 130	7.4 (1.2) n = 747	7.1 (1.2) n = 578	7.3 (1.2) n = 603	7.1 (1.2) n = 693	0.941
Total Cholesterol (mg/dL)	166.9 (1.2) n = 795	163.6 (1.2) n = 771	165.5 (1.3) n = 772	165.1 (1.3) n = 837	164.7 (1.3) n = 718	160.7 (1.3) n = 830	162.2 (1.2) n = 725	161.5 (1.2) n = 502	159.0 (1.3) n = 476	162.2 (1.3) n = 558	0.660
HDL-C (mg/dL)	41.4 (1.3) n = 660	41.9 (1.3) n = 619	42.0 (1.3) n = 620	42.6 (1.3) n = 679	42.8 (1.2) n = 568	43.0 (1.3) n = 710	43.4 (1.3) n = 649	44.5 (1.3) n = 313	44.5 (1.3) n = 621	45.2 (1.3) n = 570	0.102
LDL-C (mg/dL)	88.6 (1.4) n = 654	86.3 (1.4) n = 611	86.1 (1.5) n = 617	87.8 (1.5) n = 679	95.3 (1.4) n = 566	93.9 (1.4) n = 704	91.4 (1.4) n = 645	93.1 (1.5) n = 318	95.5 (1.4) n = 308	90.4 (1.4) n = 567	0.368
Triglycerides (mg/dL)	151.9 (1.6) n = 660	142.2 (1.6) n = 622	151.8 (1.6) n = 628	144.4 (1.6) n = 690	149.1 (1.6) n = 574	147.6 (1.7) n = 712	151.6 (1.7) n = 650	159.7 (1.7) n = 316	150.6 (1.7) n = 603	149.9 (1.6) n = 570	0.834
Systolic blood pressure (mmHg)	123.2 (1.1) n = 681	122.8 (1.1) n = 670	122.8 (1.1) n = 684	122 (1.1) n = 455	119.9 (1.1) n = 237	120.7 (1.1) n = 147	122.5 (1.1) n = 523	122.3 (1.1) n = 610	124.0 (1.1) n = 777	122.8 (1.1) n = 799	0.799
Diastolic blood pressure (mmHg)	74.3 (1.1) n = 683	73.8 (1.1) n = 670	74.0 (1.1) n = 685	72.2 (1.1) n = 456	71.6 (1.1) n = 236	70.8 (1.1) n = 148	75.1 (1.1) n = 512	74.9 (1.1) n = 609	74.7 (1.1) n = 776	73.3 (1.1) n = 799	0.133
Body mass index (kg/m^2^)	31.4 (1.2) n = 383	31.1 (1.2) n = 395	31.2 (1.2) n = 486	31.0 (1.2) n = 818	31.2 (1.2) n = 736	31.1 (1.2) n = 148	31.1 (1.2) n = 196	30.2 (1.2) n = 297	30.4 (1.2) n = 489	30.3 (1.2) n = 678	0.636*

A p-value was obtained through repeated measures ANOVA or through Friedman test (*) depending on normality. A p-value < 0.05 was considered significant. Post HOC analysis using the Sidak method denoted by 'ab. Variables with the same letter indicate no significant difference, while variables with different letters indicate a significant difference

**Fig. 1:**
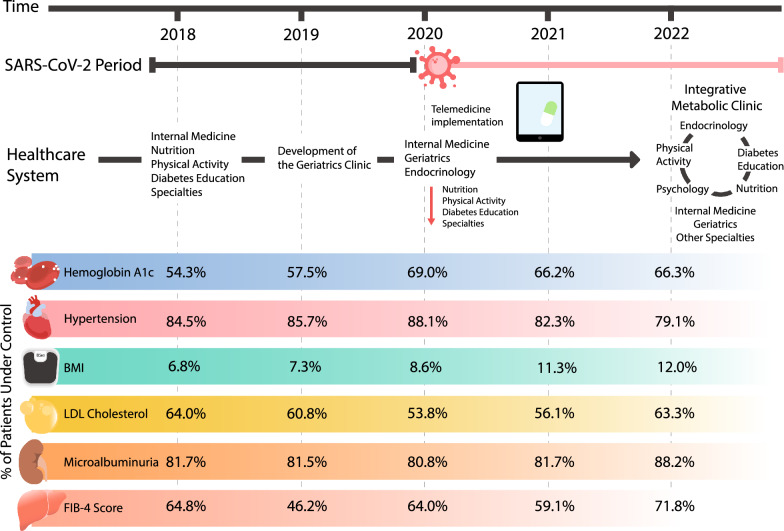
Proportion of subjects in metabolic control from 2018 to 2022. Glycated Hemoglobin considered adequately controlled when < 7%, < 7.5% in adults over 65, and < 8% in adults over 65 with multiple comorbidities, as per ADA 2024. Systolic blood pressure deemed in adequate control when < 130 mmHg, according to ADA 2024. BMI considered in control when between 18.5 and 24.9 kg/m^2^. LDL-C is considered in adequate control when levels are < 100 mg/dL, in accordance with ADA 2024. Albuminuria is considered negative when levels are < 30 mg/g, as per ADA 2024. FIB-4 Classification considered in control when classified within the category F0–F1

However, when analyzing the proportion of patients meeting control targets, HbA1c had its highest control peak at the end of 2020 at 73.8% and its minimum at the end of 2018 at 53.2%, p < 0.001. The proportion of patients with controlled FG was at its minimum in 2018 (13.8%); with its highest peak at the end of 2021 (53.8%), p < 0.001. Regarding systolic blood pressure values, there was a peak control proportion in 2020 at 89.8%, which subsequently remained within the range of > 80%, with a statistically significant p-value of < 0.001. As for diastolic blood pressure, the peak proportion of controlled patients was observed in late 2018 (93%), with the lowest point occurring in early 2022 at 88.1%, p = 0.01. Regarding HDL-C, its highest control proportion occurred at the beginning of 2022 with 87%, and its lowest control proportion was at the end of the same year with p < 0.001. The proportion of subjects in LDL-C control peaked in 2022 at 69.7% and its minimum in 2020 at 52.7%, p < 0.001. The proportion of subjects in triglyceride control remained > 50% for the most part, only reaching its lowest point at the end of 2021 with 47.5% and its highest point at the end of 2019 (57.1%), p = 0.161. Concerning albuminuria, 89% of patients had adequate control at the end of 2022 and its lowest control proportion at the end of 2020 with 75.4% of patients, p = 0.002. When analyzing the FIB-4 classification, there was an increase in the patients classified as F0–F1 in 2018; 64.8% of patients were observed in the F0–F1 classification; this remained similar in 2020, with 64.0% of patients, and subsequently increased to 71.8% in 2022, p < 0.001. Regarding those classified as F2, the highest proportion of patients in this classification was observed in 2019 at 42.8%, followed by its lowest proportion in 2022 at 20.9% (p < 0.001). In the case of patients classified as F3-F4, there was a notable variation over the years, with the lowest proportion in 2018 at 4.9%, subsequently increasing in 2019 to 11.0% of patients, and gradually decreasing again, with 8.3% of patients classified as F3-F4 in 2021, and further decreasing in 2022 to 7.3% of patients, p < 0.001. Regarding BMI classification, proportion of controlled patients varied between 6 and 14%, reaching its highest peak at the end of 2021 and its lowest peak at the beginning of 2018, p < 0.001. The proportion of patients under control based on laboratory parameters is detailed in Fig. 1 and Table 2.

**Table 2:** Proportion of controlled patients based on goals from 2018 to 2022

Parameter in control	Beginning 2018 n(%)	End of 2018 n(%)	Beginning 2019 n(%)	End of 2019 n(%)	Beginning 2020 n(%)	End of 2020 n(%)	Beginning 2021 n(%)	End of 2021 n(%)	Beginning 2022 n(%)	End of 2022 n(%)	p-value^1^
Fasting Serum Glucose	110 (13.8) n = 796	126 (16.3) n = 775	488 (56.2) n = 869	491 (55.2) n = 889	421 (54.8) n = 768	476 (56.5) n = 843	455 (54.6) n = 833	285 (59.5) n = 479	378 (50.6) n = 747	407 (56.1) n = 726	< 0.001
Glycated Hemoglobin	493 (55.5) n = 888	308 (53.2) n = 579	171 (53.8) n = 318	586 (61.3) n = 956	261 (64.3) n = 406	96 (73.8) n = 130	461 (61.7) n = 747	410 (70.8) n = 579	373 (61.9) n = 603	490 (70.7) n = 693	< 0.001
HDL-C	415 (62.9) n = 660	372 (60.1) n = 619	379 (61.1) n = 620	401 (59.1) n = 679	325 (57.2) n = 568	412 (58) n = 710	361 (55.6) n = 649	157 (50.2) n = 313	689 (87.0) n = 792	277 (48.6) n = 570	< 0.001
LDL-C	406 (62.1) n = 654	403 (66.0) n = 611	384 (62.2) n = 617	403 (59.4) n = 679	298 (52.7) n = 566	387 (55.0) n = 704	385 (59.0) n = 653	173 (53.2) n = 325	476 (69.7) n = 683	326 (57.0) n = 572	< 0.001
Triglycerides	356 (53.9) n = 660	354 (56.9) n = 622	338 (53.8) n = 628	394 (57.1) n = 690	305 (53.1) n = 574	389 (54.6) n = 712	333 (51.2) n = 650	150 (47.5) n = 316	322 (53.4) n = 603	299 (52.5) n = 570	0.161
Negative Albuminuria	64 (80.5) n = 74	263 (83.0) n = 317	247 (81.0) n = 305	316 (82.1) n = 385	208 (86.3) n = 241	230 (75.4) n = 305	266 (82.4) n = 323	47 (81.0) n = 58	250 (87.4) n = 683	219 (89.0) n = 246	0.002
FIB-4 0–1 Classification	199 (65)	n = 307	67 (46)	n = 145	169 (64)	n = 264	234 (59)	n = 396	79 (72)	n = 110	< 0.001
FIB-4 2 Classification	93 (30)	n = 307	62 (43)	n = 145	82 (31)	n = 264	129 (33)	n = 396	23 (21)	n = 110	< 0.001
FIB-4 3–4 Classification	15 (4.9)	n = 307	16 (11)	n = 145	13 (4.9)	n = 264	33 (8.3)	n = 396	8 (7.3)	n = 110	< 0.001
Systolic blood pressure	563 (82.7) n = 681	559 (83.4) n = 670	580 (84.8) n = 684	394 (86.6) n = 455	212 (89.8) n = 236	127 (86.4) n = 147	424 (81.1) n = 523	505 (82.8) n = 610	607 (78.1) n = 777	640 (80.1) n = 799	< 0.001
Diastolic blood pressure	612 (89.6) n = 683	623 (93) n = 670	625 (91.2) n = 685	417 (91.4) n = 456	211 (89.4) n = 236	135 (91.2) n = 148	446 (87.1) n = 512	684 (88.1) n = 776	684 (88.1) n = 776	729 (91.4) n = 798	0.01
Body mass index	23 (6.0) n = 383	30 (7.6) n = 395	34 (7.0) n = 486	62 (7.6) n = 818	53 (7.2) n = 736	15 (10.1) n = 148	17 (8.7) n = 196	43 (14) n = 297	57 (12) n = 489	80 (12) n = 678	< 0.001

^1^p-value obtained by Chi square. A p-value < 0.05 was considered significant. Fasting serum glucose considered in control when within the range of 80—130 mg/dL. Glycated Hemoglobin adequately controlled when < 7%, < 7.5% in adults over 65, and < 8% in adults over 65 with multiple comorbidities. HDL-C considered in adequate control when levels are < 50 mg/dL in women and < 40 mg/dL in men. LDL-C is considered in adequate control when levels are < 100 mg/dL. Triglycerides is considered in adequate control when levels are < 150 mg/dL. Albuminuria is considered negative when levels are < 30 mg/g. FIB-4 is considered in control when classified within the category F0–F1. Systolic blood pressure is considered in adequate control when < 130 mmHg, and diastolic blood pressure is considered in adequate control when < 80 mmHg. BMI considered in adequate control when between 18.5 and 24.9 kg/m^2^

The AUC of the difference in the mean values of metabolic parameters over time was computed and compared between subjects infected by SARS-CoV-2 and those who did not have the infection. Results showed statistically higher AUC values in subjects with SARS-CoV2 in the following parameters: FG, total cholesterol, LDL-C, triglycerides, diastolic blood pressure, and BMI (Table 3).

**Table 3:** The area under the curve of the mean of metabolic parameters during 5 years of follow-up

AUC Parameter Mean (SD)^1^	No SARS-CoV-2 (n = 603)	SARS-CoV-2 (n = 396)	**p-value**^**3**^
Fasting glucose	508 (143)	532 (125)	0.008
Glycated hemoglobin	27 (8)	28 (6)	0.13
Total Cholesterol	581 (167)	626 (139)	< 0.001
HDL-C	154 (48)	161 (44)	0.061
LDL-C	323 (113)	344 (106)	0.001
Triglycerides	581 (366)	613 (341)	0.025
Systoilc Pressure	445 (101)	446 (99)	> 0.9
Dyastolic Pressure	267 (62)	276 (57)	0.007
BMI ^2^	95 (34)	103 (33)	0.003

^1^AUC = area under the curve, ^2^BMI = Body Mass Index, ^3^Mann–Whitney U test

Regarding consultation type, telephone consultations were implemented in 2020, with 899 (90%) patients using this modality at least once, reducing in-person consultations, with only 541 (54.2%) patients using this modality. In-person consultations reached their lowest point in 2021, with 2 (0.2%) patients. All patients attended at least one in-person or telephonic follow-up consultation with internal medicine or geriatrics during the years of this study. Ophthalmology consultations varied significantly, with the highest attendance in 2019 (50.8%) and its lowest during 2020 (28.5%), p < 0.001. Retinology consultations attendance peaked in 2018 with 56 (5.6%) patients and gradually reduced to reach its lowest attendance in 2022 with only 26 (2.6%) patients. Attendance to endocrinology consultations increased over the years, with its minimum attendance in 2018 with 85 (8.5%) patients and subsequently reaching its highest attendance proportion with 121 (12.1%) patients in 2022; similarly, cardiology consultations had its lowest peak of attendance in 2018 with 69 (6.9%) patients and reached maximum attendance with 103 (10.3%) patients in 2022. Diabetes education consultations peaked in 2018 with 274 (27.4%) patients, mirroring nutrition and physical activation consultations, reaching their highest point with 124 (12.4%) patients in the same year. Table 4 displays the number of patients attending each consultation and their respective percentages each year.

**Table 4:** Consultation attendance during 2018 and 2022

Consultation modality	2018 n = 999 (%)	2019 n = 999 (%)	2020 n = 999 (%)	2021 n = 999 (%)	2022 n = 999 (%)	p-value
Telemedicine	0 (0)	0 (0)	105 (10.5)	5 (0.5)	5 (0.5)	< 0.001
Telephonic	0 (0)	899 (90)	899 (90.0)	999 (100)	999 (100)	< 0.001
In-person	999 (100)	541 (54.2)	541 (54.2)	2 (0.2)	278 (27.8)	< 0.001
Specialty consultation attendance during 2018 and 2022						
Specialty	2018 n = 999 (%)	2019 n = 999 (%)	2020 n = 999 (%)	2021 n = 999 (%)	2022 n = 999 (%)	p-value
Cardiology	69 (6.9)	101 (10.1)	83 (8.3)	85 (8.5)	103 (10.3)	0.006
Diabetes' Education	274 (27.4)	246 (24.6)	125 (12.5)	72 (7.2)	90 (9.0)	< 0.001
Endocrinology	85 (8.5)	91 (9.1)	99 (9.9)	108 (10.8)	121 (12.1)	< 0.001
Nephrology	29 (2.9)	33 (3.3)	34 (3.4)	40 (4.0)	38 (3.8)	0.342
Neurology	50 (5.0)	49 (4.9)	35 (3.5)	42 (4.2)	33 (3.3)	0.103
Nutrition/physical activation	124 (12.4)	108 (10.8)	56 (5.6)	81 (8.1)	63 (6.3)	< 0.001
Ophthalmology	474 (47.4)	507 (50.8)	285 (28.5)	355 (35.5)	369 (36.9)	< 0.001
Retinology	56 (5.6)	55 (5.5)	37 (3.7)	33 (3.3)	26 (2.6)	< 0.001

p-value obtained through the Cochran's Q test. p-value < 0.05 was considered statistically significant. Internal Medicine and Geriatrics consultation for all patients based on the patient's age. From 2018 to 2020, patients aged over 70 years for Geriatrics consultation, and from 2021 onwards, patients over 65 years for Geriatrics consultation

Regarding medication usage and prescription, there was an overall increase in oral hypoglycemic agents. Prescription of biguanides increased gradually from 853 (85.8%) patients in 2018 to 902 (90,7%) patients in 2020 and proceeded to decrease to 805 (80.6%) patients in 2022, p < 0.001. Contrary to the rest, sulfonylureas usage decreased over the study years, with its maximum prescription peak in 2018 with 143 (14.4%) patients and having its lowest peak in 2022 with 62 (6.2%) patients, p < 0.001. In relation to insulin use, long-lasting insulin was the most prescribed with 371 (37.1) patients, with a gradual increase of usage from 182 (18.3%) patients in 2018, to 243 (24.3%) patients in 2022, p < 0.001.

Regarding lipid-lowering medications, 81.9% of patients are using statins, which reached their peak in 2020 with 619 (62.2%) patients using this medication. On the other hand, 273 (27.3%) patients used fibrates, with their highest usage in 2018 being 147 (14.8%) patients, and there was a gradual reduction in their use over time. In terms of the use of lipid-lowering medications, 81.9% of patients have used statins, reaching their highest point in 2020 with 619 (62.2%) patients using this medication.

On the other hand, 273 (27.3%) patients used fibrates, with its highest usage in 2018 with 147 (14.8%) patients and a gradual reduction in their prescription over time. Among the antihypertensive medications, angiotensin II receptor blockers were the most used, with 504 (50.5%) patients. Table 5 shows the most frequently prescribed medications.

**Table 5:** Medication group used by patients for control of diabetes mellitus and comorbidities from 2018 to 2022

Medication group	2018 n = 999 (%)	2019 n = 999 (%)	2020 n = 999 (%)	2021 n = 999 (%)	2022 n = 999 (%)	Entre 2018 y 2022 n = 999 (%)	p-value
Hypoglycemic agents							
Biguanides	853 (85.8)	891 (89.5)	902 (90.7)	809 (81.0)	805 (80.6)	969 (97.0)	< 0.001
GLP-1 analogs	33 (3.3)	80 (8.0)	93 (9.3)	95 (9.5)	88 (8.8)	176 (17.6)	< 0.001
DPP-4 inhibitors	572 (57.5)	660 (66.3)	673 (67.6)	602 (60.3)	614 (61.5)	876 (87.7)	< 0.001
SGLT2 inhibitors	265 (26.7)	328 (33.0)	407 (40.9)	417 (41.7)	443 (44.3)	650 (65.1)	< 0.001
Thiazolidinediones	88 (8.9)	142 (14.2)	161 (16.2)	152 (15.2)	158 (15.8)	318 (31.8)	< 0.001
Sulfonylureas	143 (14.4)	150 (15.1)	118 (11.9)	67 (6.7)	62 (6.2)	247 (24.7)	< 0.001
Insulins							
Long-acting insulin	182 (18.3)	210 (21.1)	242 (24.2)	237 (23.7)	243 (24.3)	371 (37.1)	< 0.001
Ultra-rapid-acting Insulin	71 (7.1)	64 (6.4)	52 (5.2)	49 (4.9)	50 (5.0)	142 (14.2)	0.065
Regular insulin	72 (7.2)	64 (6.4)	52 (5.2)	33 (3.3)	30 (3.0)	100 (10.0)	< 0.001
Ultra-long-acting insulin	0 (0)	4 (0.4)	6 (0.6)	5 (0.5)	10 (1.0)	17 (1.7)	0.010
Intermediate-acting Insulin	6 (0.6)	9 (0.9)	6 (0.6)	1 (0.1)	0 (0)	10 (1.0)	0.017
Lipid-lowering medications							
Statins	537 (54.0)	589 (59.2)	619 (62.2)	576 (57.7)	575 (57.6)	828 (81.9)	< 0.001
Fibrates	147 (14.8)	134 (13.5)	130 (13.1)	118 (11.8)	123 (12.3)	273 (27.3)	0.546
Antihypertensive medications							
Angiotensin II receptor blockers	275 (27.7)	319 (32.1)	352 (35.4)	328 (32.8)	342 (34.2)	504 (50.5)	< 0.001
Calcium channel blockers	96 (9.7)	121 (12.2)	135 (13.6)	143 (14.3)	152 (15.2)	227 (22.7)	< 0.001
Beta-blockers	104 (10.5)	121 (12.2)	113 (11.4)	99 (9.9)	110 (11.0)	197 (19.7)	0.271
Angiotensin-converting enzyme inhibitor	142 (14.3)	148 (14.9)	119 (12.0)	87 (8.7)	80 (8.0)	242 (24.2)	< 0.001
diuretics	30 (3.0)	39 (3.9)	76 (7.6)	58 (5.8)	66 (6.6)	147 (14.7)	< 0.001
Antiarrhythmics	10 (1.0)	5 (0.5)	7 (0.7)	5 (0.5)	11 (1.1)	20 (2.0)	0.206
Other medications							
NSAIDs	408 (41.0)	435 (43.7)	401 (40.3)	37 (3.7)	38 (3.8)	582 (58.3)	< 0.001
Gabapentin	28 (2.8)	22 (2.2)	20 (2.0)	23 (2.3)	21 (2.1)	59 (5.9)	0.80
pregabalin	32 (3.2)	37 (3.7)	34 (3.4)	24 (2.4)	26 (2.6)	83 (8.3)	0.48

p-value obtained through the Cochran's Q test. p-value < 0.05 was considered statistically significant

NSAIDs = Non-steroid-anti-inflammatory drugs

In reference to complications secondary to type 2 diabetes (T2D), there were no significant changes. Diabetic neuropathy [35 (3.6%)] was the most common microvascular complication, ischemic heart disease [11 (1.1%)] among macrovascular complications, and hypoglycemia [14 (1.4%)] among acute complications (Table 6).

**Table 6:** Incidence of complications secondary to diabetes mellitus in patients from 2018 to 2022

Type of complication		Between 2018 and 2022	2018 (%)	2019 (%)	2020 (%)	2021 (%)	2022 (%)	p-value
Microvascular complications	Diabetic neuropathy	35 (3.6) n = 967	4 (0.4) n = 967	7 (0.7) n = 967	6 (0.6) n = 967	15 (1.6) n = 967	7 (0.7) n = 967	0.106
	Diabetic nephropathy	22 (2.2) n = 967	5 (0.5) n = 967	2 (0.2) n = 967	6 (0.6) n = 967	7 (0.7) n = 967	3 (0.3) n = 967	0.442
	Diabetic retinopathy	16 (1.7) n = 967	3 (0.3) n = 967	3 (0.3) n = 967	1 (0.1) n = 967	3 (0.3) n = 967	5 (0.5) n = 967	0.615
	Diabetic foot	5 (0.5) n = 967	1 (0.1) n = 967	2 (0.2) n = 967	0 (0) n = 967	1 (0.1) n = 967	0 (0) n = 967	0.478
Acute complications	Hypoglycemia	14 (1.4) n = 967	2 (0.2) n = 967	3 (0.3) n = 964	5 (0.5) n = 967	4 (0.4) n = 967	3 (0.3) n = 967	0.804
	Hyperosmolar hyperglycemic state	6 (0.6) n = 967	0 (0) n = 967	1 (0.1) n = 964	2 (0.2) n = 967	1 (0.1) n = 967	2 (0.2) n = 967	0.675
	Diabetic ketoacidosis	2 (0.2) n = 967	0 (0) n = 967	0 (0) n = 964	0 (0) n = 967	0 (0) n = 967	1 (0.1) n = 967	0.406
Macrovascular complications	Ischemic heart disease	11 (1.1) n = 967	2 (0.2) n = 967	1 (0.1) n = 967	2 (0.2) n = 967	4 (0.4) n = 967	2 (0.2) n = 967	0.702
	Ischemic stroke	4 (0.4) n = 967	1 (0.1) n = 967	1 (0.1) n = 967	0 (0) n = 967	1 (0.1) n = 967	1 (0.1) n = 967	0.91

A p-value was obtained through the Cochran's Q test. A p-value < 0.05 was considered statistically significant

## Discussion

This study focuses on a cohort of 999 patients with T2D patients treated between 2018 and 2022 at a hospital in Northeastern Mexico, where a multidisciplinary team navigated challenges and adaptations during the SARS-CoV-2 pandemic. It was observed that, despite a decrease in in-person consultations and an increase in telemedicine, significant improvements in glycemic control were observed. HbA1c based control proportion was lower before the pandemic and improved in subsequent years. A significant decrease in FG was observed during 2019–2022 compared to 2018, while levels of HbA1c and cholesterol showed no significant changes. The proportion of patients with BMI at goal increased in 2022, and blood pressure control was maintained. The proportion of FIB-4 score 0–1 increased over time. On the other hand, there was a decrease in the proportion of patients with controlled LDL-C during the years in which the pandemic unfolded. Additionally, when the AUC of the metabolic control was compared between subjects that had SARS-CoV-2 infection vs. those that did not, there were higher AUC values in subjects that had SARS-CoV2 in fasting glucose, total cholesterol, LDL-C, triglycerides, diastolic blood pressure, and BMI.

### Demographics and proportion of metabolic control parameters

Our population was predominantly male, which aligns with a study of 500,000 patients describing a higher prevalence of type 2 diabetes in male patients of various ethnicities compared to the female population [13]. This predominance of the male population may be related to the diagnosis of diabetes occurring earlier in men than in women, especially between the ages of 35 and 69, consistent with our study’s average age of 60 years [14].

In our population, the proportion of controlled patients based on HbA1c remained in a high range between 53.2 and 73.8%. This contrasts with a study conducted in Mexico City (1998–2005, 2015–2019), which reported a lower proportion with good control between 16 and 37% [15]. Similarly, another study conducted by the National Health and Nutrition Examination Survey from 1999 to 2010 reported a control improvement until early 2010, with a subsequent stall and declination to 50.5% in 2018 [16]. Our controlled proportion was higher and even increased in the following years compared to other populations.

Throughout the study, the proportion of patients with HbA1c levels in control showed an increase and subsequent stabilization in 2020 and 2022 compared to previous years. This contrasts with a meta-analysis from 21 studies, which demonstrated a significant deterioration in HbA1c levels and a decrease in the control proportion during the SARS-COV-2 pandemic compared to previous years [5]. In another study from the U.S. evaluating patients between 1999 and 2018, there was a decrease in the proportion of control, from 57.4 to 50.5% during 2002–2010 [16], even without a pandemic. Our patient’s improvement in control is related to their access to a multidisciplinary team. Despite the reduction of in-person visits to telephone consultations, key diabetes-related specialties remained available for patient care. This is consistent with various studies that state that a multidisciplinary team composed of specialists in the medical field, nutrition, physical activity, and diabetes education has a beneficial effect on reducing HbA1c, FG, and blood pressure levels through constant monitoring, medical guidance, and appropriate treatment, which is consistent with the practices of the metabolic clinic at the hospital [17, 18].

Despite transitioning from traditional in-person consultations to telephonic medical care in 2020, glycemic control improved and stabilized during the pandemic compared to previous years. Similar to our research, a study from Louisiana evaluated the effectiveness of telemedicine in controlling the HbA1c levels during the SARS-COV-2 pandemic, showing a decrease in these levels during the pandemic period [19]. Likewise, a study conducted in Japan in 2019 and 2020, where telemedicine consultations were implemented, found that patients who were within control range prior to the pandemic maintained stable control [20].

A difference in the control proportion of LDL-C levels was observed, with a more significant decrease in 2020. This may correlate with the reduced attendance to nutrition and physical activity consultations due to the prioritization of internal medicine and endocrinology for patient management during the pandemic. Similarly, due to the pandemic and subsequent lockdown, accessibility and availability of food were affected, disrupting the quality of the diet [21], leading to an increase in the consumption of ultra-processed foods and alcohol, and a decreased intake of fruits, vegetables, and whole grains, as well as a decline in physical activity [22, 23].

Analyzing the FIB-4 classification, an increase in patients classified as F0–F1 was observed, with a consequent decrease in those classified as F2. This contrasts with a study that analyzed the trend of hepatic fibrosis in 3 years of 1527 patients with T2D, which showed a decrease in patients with low risk of fibrosis and an increase of intermediate and high-risk patients during the three-year follow-up [24]. This could relate to obesity and high BMI’s association with the development of Metabolic Dysfunction-Associated Liver Disease (MASLD), secondary to the production of proinflammatory cytokines by the adipose tissue that triggers the progression of MASLD [25]. In our population, the higher proportion of low-risk FIB-4 was in 2022, which coincides with the rise of patients with healthy BMI levels in 2022.

### SARS-CoV-2 and effects on metabolic control parameters

During the study, we ensured that patient metabolic parameters were not evaluated during acute infection phases. When comparing AUC between subjects with a SARS-CoV-2 infection at any point vs. those without, the former exhibited higher AUC levels of fasting glucose, total cholesterol, LDL-C, diastolic blood pressure, and BMI. This suggests that SARS-CoV-2 could have a deleterious effect on metabolic control for the long term. However, there could also be a bidirectional relation since subjects with poor metabolic control can be more prone to SARS-CoV-2 infection.

In relation to SARS-CoV-2 and glucose metabolism, pre-existing T2D can exacerbate SARS-CoV-2 infection. High glucose promotes SARS-CoV-2 entry by upregulating ACE2 expression. The virus can also impair islet function by directly infecting pancreatic beta cells, restricting insulin secretion, and inducing cell apoptosis. Additionally, SARS-CoV-2 infection can shift glucose metabolism towards aerobic glycolysis through the Warburg effect [26].

Lipid metabolism is indispensable for providing energy, maintaining homeostasis, and regulating the immune response. Lipid droplets have been reported to be associated with antiviral innate immunity. Lipid accumulation has been observed in the lungs of SARS-CoV-2 patients. Furthermore, significant lipidomic alterations have been linked to SARS-CoV-2 severity since fatty acids are essential for SARS-CoV-2 replication [26].

SARS-CoV-2 specifically recognizes and attaches human angiotensin-converting enzyme 2 (ACE2) for entry via the S protein. Different ACE2 polymorphisms, such as rs2074192, have been associated with hypertension in obese males. Additionally, previous studies have shown a higher incidence of hypertension post- SARS-CoV-2 infection [28].

The relation between higher BMI and SARS-CoV-2 infection can be explained through multiple mechanisms, such as altered respiratory anatomy related to fat deposits in the mediastinum and abdomen and reduced chest wall elasticity. Also, obese subjects have an imbalance of coagulant factors, increased leptin resistance, B cell and T cell impairment, complement system overreaction, and poor antibody response. Adipose tissue, serving as a viral reservoir, could prolong virus shedding in obese patients and increase viral replication [29].

### Visits to medical specialties

Regarding consultations, all patients are under close monitoring by an internist or geriatrician depending on their age, aiming for adherence to the ADA 2024 guidelines for continuous monitoring of patients with T2D [30]. There was a decrease in the assistance to ophthalmology, diabetes education, nutrition, and physical activity consultations, while endocrinology and cardiology consultations increased. Nephrology and neurology consultations remained unchanged.

There was a decrease in attendance at ophthalmology consultations. According to the ADA 2024 guidelines, it is recommended that all patients with T2D have a comprehensive eye examination by an ophthalmologist at the time of diagnosis and annually if there are signs of retinopathy. However, when there is no evidence of retinopathy in one or more annual eye exams and glucose parameters are under control, screening tests can be conducted every 2 years [31]. Due to the low incidence of diabetic retinopathy in our population compared to others [32, 33] and patients maintaining controlled glucose levels, the less frequent referral to ophthalmology can be justified, leading to a decrease in attendance for these consultations.

The ADA does not provide a specific recommendation for a minimum number of endocrinology consultations for patients with T2D; however, it emphasizes the value of a multidisciplinary team [31]. At the onset of the pandemic, there was an increase in attendance for endocrinology and cardiology consultations, which can be interpreted as an effort to maintain and increase the proportion of patients in control based on HbA1c, FG, and lipid levels, as well as a compensatory response to the decreased attendance in diabetes education, nutrition, and physical activity consultations. Like our population, a study mentions that patients with well-controlled T2D can receive adequate care through an internist, nutritionist, and diabetes educator. It also states that consultations with an endocrinologist are reserved for the care and treatment of more complex patients [34].

There was no significant change in attendance for nephrology and neurology consultations. This is because, referral to these specialties should be considered in specific situations of greater complexity [11]. Diabetic nephropathy and neuropathy are the most common complications in our population over the 5-year period, which explains the consistent attendance for these consultations.

### T2D management

Prescription of oral hypoglycemic agents increased between 2019 and 2022, except for sulfonylureas, which decreased in this same period. This finding aligns with a study conducted in Germany that analyzed the change in glucose-lowering regimens during the SARS-CoV-2 pandemic, showing an increased prescription of different oral hypoglycemic agents such as biguanides, DPP-4 inhibitor, SGLT2 inhibitors and GLP-1 analogs between 2019 and 2020 [35], with a higher increase in SGLT2 inhibitors, same as our study. This can be related to its positive outcomes regarding glycemic control and other beneficial effects, such as cardiovascular protection, blood pressure reduction, and improved kidney function, which could have been beneficial during the pandemic as an additional vital organ protection [36].

Between 2018 and 2022, there was an increase in the prescription of oral hypoglycemic agents and insulin and a decrease in sulfonylureas. This coincides with research conducted in Mexico City that analyzed trends in management from 1998 to 2004 and from 2015 to 2019. Said study found a general increase in glucose-lowering medication, a moderate increase in insulin use, and a decrease in sulfonylureas [15]. The increase in the prescription of hypoglycemic agents can be attributed to the progressive nature of type 2 diabetes, necessitating combined therapies for maintaining glycemic targets. The ADA 2024 recommends the prescription of an additional medication to metformin to maintain HbA1c targets [37] which explains the increase of all hypoglycemic agents and long-acting insulin prescription. Conversely, while sulfonylureas persist as one of the most prescribed second-line agents, their usage is diminishing due to the emergence of new, beneficial, and safer treatment options for patients [38].

### Incidence of micro and macrovascular complications

Our population showed a low incidence of complications over the 5-year study compared to other populations. A study conducted in India reported that at least 46% of its population had some complication secondary to T2D [39]. Another study in Arabia reported an incidence of 76% of its population with T2D-associated complications [40]. The low occurrence of complications in our population relates to stable levels of HbA1c and FG, as chronic hyperglycemia is a significant factor in the development of microvascular complications, secondary to the impairment of capillary microvasculature by the activity of the polyol pathway [41].

Our study found a higher proportion of microvascular complications compared to macrovascular ones, with diabetic neuropathy being the most frequent complication in our population. This relates to a study conducted in Asia, which identified a higher prevalence of microvascular complications and a greater prevalence of diabetic neuropathy [42]. Similarly, a U.S. study analyzing patients from various ethnic backgrounds reported a higher incidence of diabetic neuropathy, followed by nephropathy, aligning with our results [43]. The higher prevalence of this complication is linked to an increased risk of diabetic neuropathy with advancing age. Age greater than 60 years is associated with a heightened risk of developing diabetic neuropathy due to biological aging processes such as alterations in nerve vasculature, increased advanced glycation end products, and decreased resistance to oxidative stress products [44].

### Relevance and limitations

The relevance of this study relies on its tight 5-year follow-up of a cohort of 999 patients from a poorly studied population managed by an adaptative multidisciplinary team. It demonstrated adequate glycemic control, even during the SARS-COV-2 pandemic, along with a low rate of complications compared to other studied populations.

This study has various limitations; the main limitation lies in its retrospective nature, which limits patients who had a follow-up through medical records. The assessment of the cohort was not uniform; due to the nature of the SARS-CoV-2 pandemic, some subjects did not have an evaluation at all time points. Additionally, the sample may not be representative of the general population as it reflects a population managed by a specific healthcare system in a Northeast Mexican region; however, if this health system model is replicated in other regions, it could aid in diabetes control. For future research, it would be relevant to address risk factors influencing patients' metabolic control, such as lifestyle changes and psychosocial factors. This could contribute to the development of strategies for prevention and intervention in future situations like the SARS-COV-2 pandemic.

## Conclusions

In conclusion, the findings of this research underscore the importance of an adaptative interdisciplinary approach in the care of patients with T2D. Despite the changing dynamics of healthcare during 2018–2022, due to the SARS-COV-2 pandemic, an increase in the proportion of patients with glycemic control was observed. This coincided with the implementation of telemedicine, an increase in the prescription of hypoglycemic agents, and an increase in consultations with endocrinology and cardiology. However, the decrease in the proportion of patients with controlled LDL-C emphasizes the need for comprehensive care addressing cardiovascular risk factors. When comparing AUC metabolic control between subjects with SARS-CoV2 infection vs. those that did not have the infection, there was higher FG, Total cholesterol, LDL-C, triglycerides, diastolic blood pressure, and BMI, suggesting a bidirectional effect of subjects with comorbidities and SARS-CoV-2 infection. The stability in the occurrence of complications suggests that the interdisciplinary approach may have contributed to maintaining overall good disease control. These results highlight the importance of coordinating care across different medical specialties to optimize T2D management.

## Data Availability

The datasets used and/or analyzed during the current study are available from the corresponding author on reasonable request.

## Abbreviations

ACE2: Angiotensin-converting enzyme 2
ANOVA: Analysis of variance
ADA: American Diabetes Association
AUC: Area under the curve
BMI: Body mass index
BP: Blood pressure
DPP-4: Dipeptidyl Peptidase 4
FG: Fasting serum glucose
FIB-4: Fibrosis 4 Index for liver fibrosis
GLP-1: Glucagon-like peptide
HbA1c: Glycated hemoglobin
HDL-C: High-density lipoprotein cholesterol
LDL-C: Low-density lipoprotein cholesterol
MASLD: Metabolic Dysfunction-Associated Liver Disease
SARS-CoV-2: Severe acute Respiratory Syndrome Coronavirus 2
SD: Standard deviation
SGLT-2: Sodium glucose co-transporter 2
NSAIDs: Non-steroid-anti-inflammatory drugs
STROBE: Strengthening the Reporting of Observational Studies in Epidemiology
T2D: Type 2 diabetes mellitus

## References

[1] American Diabetes Association Professional Practice Committee, ElSayed NA, Aleppo G, Bannuru RR, Bruemmer D, Collins BS, et al. 4. Comprehensive Medical Evaluation and Assessment of Comorbidities: *Standards of Care in Diabetes—2024*. Diabetes Care. 2024;47(Supplement_1):S52–76. 10.2337/dc24-S004 PMC10725809 38078591

[2] Bello-Chavolla OY, Antonio-Villa NE, Fermín-Martínez CA, Fernández-Chirino L, Vargas-Vázquez A, Ramírez-García D (2022). Diabetes-related excess mortality in mexico: a comparative analysis of national death registries between 2017–2019 and 2020. Diabetes Care.

[3] Cheikh Ismail L, Osaili TM, Mohamad MN, Al Marzouqi A, Jarrar AH, Abu Jamous DO (2020). Eating habits and lifestyle during COVID-19 lockdown in the United Arab Emirates: a cross-sectional study. Nutrients.

[4] Kendzerska T, Zhu DT, Gershon AS, Edwards JD, Peixoto C, Robillard R (2021). The Effects of the Health System Response to the COVID-19 pandemic on chronic disease management: a narrative review. Risk Manag Healthc Policy.

[5] Wafa IA, Pratama NR, Sofia NF, Anastasia ES, Konstantin T, Wijaya MA (2022). Impact of COVID-19 lockdown on the metabolic control parameters in patients with diabetes mellitus: a systematic review and meta-analysis. Diabetes Metab J.

[6] Herrington WG, Alegre-Díaz J, Wade R, Gnatiuc L, Ramirez-Reyes R, Hill M (2018). Effect of diabetes duration and glycaemic control on 14-year cause-specific mortality in Mexican adults: a blood-based prospective cohort study. Lancet Diabetes Endocrinol.

[7] Cuschieri S (2019). The STROBE guidelines. Saudi J Anaesth.

[8] American Diabetes Association Professional Practice Committee, ElSayed NA, Aleppo G, Bannuru RR, Bruemmer D, Collins BS, et al. 6. Glycemic Goals and Hypoglycemia: *Standards of Care in Diabetes—2024*. Diabetes Care. 2024;47(Supplement_1):S111–25. 10.2337/dc24-S006 PMC10725808 38078586

[9] American Diabetes Association Professional Practice Committee, ElSayed NA, Aleppo G, Bannuru RR, Bruemmer D, Collins BS, et al. 13. Older Adults: *Standards of Care in Diabetes—2024*. Diabetes Care. 2024;47(Supplement_1):S244–57. 10.2337/dc24-S013 PMC10725804 38078580

[10] American Diabetes Association Professional Practice Committee, ElSayed NA, Aleppo G, Bannuru RR, Bruemmer D, Collins BS, et al. 10. Cardiovascular Disease and Risk Management: *Standards of Care in Diabetes—2024*. Diabetes Care. 2024;47(Supplement_1):S179–218. 10.2337/dc24-S010 PMC10725811 38078592

[11] American Diabetes Association Professional Practice Committee, ElSayed NA, Aleppo G, Bannuru RR, Bruemmer D, Collins BS, et al. 11. Chronic Kidney Disease and Risk Management: *Standards of Care in Diabetes—2024*. Diabetes Care. 2024;47(Supplement_1):S219–30. 10.2337/dc24-S011 PMC10725805 38078574

[12] Centers For Disease Control and Prevention. About Adult BMI. 2022. https://www.cdc.gov/healthyweight/assessing/bmi/adult_bmi/index.html

[13] Ferguson LD, Ntuk UE, Celis-Morales C, Mackay DF, Pell JP, Gill JMR (2018). Men across a range of ethnicities have a higher prevalence of diabetes: findings from a cross-sectional study of 500 000 UK Biobank participants. Diabet Med.

[14] Tramunt B, Smati S, Grandgeorge N, Lenfant F, Arnal JF, Montagner A (2020). Sex differences in metabolic regulation and diabetes susceptibility. Diabetologia.

[15] Aguilar-Ramirez D, Alegre-Díaz J, Gnatiuc L, Ramirez-Reyes R, Wade R, Hill M (2021). Changes in the diagnosis and management of diabetes in mexico city between 1998–2004 and 2015–2019. Diabetes Care.

[16] Fang M, Wang D, Coresh J, Selvin E (2021). Trends in Diabetes Treatment and Control in US Adults, 1999–2018. N Engl J Med..

[17] Gabay L, Menashe I, Yehoshua I (2019). 689-P: the effect of multidisciplinary intervention on diabetes control in patients with poor glycemic control. Diabetes..

[18] Siaw MYL, Lee JYC (2019). Multidisciplinary collaborative care in the management of patients with uncontrolled diabetes: a systematic review and meta-analysis. Int J Clin Pract.

[19] Walker B, Stoecker C, Shao Y, Nauman E, Fort D, Shi L (2023). Telehealth and medicare type 2 diabetes care outcomes: evidence from Louisiana. Med Care.

[20] Onishi Y, Ichihashi R, Yoshida Y, Tahara T, Kikuchi T, Kobori T (2022). Substitution of telemedicine for clinic visit during the COVID -19 pandemic of 2020: comparison of telemedicine and clinic visit. J Diabetes Investig.

[21] Scarmozzino F, Visioli F (2020). Covid-19 and the subsequent lockdown modified dietary habits of almost half the population in an italian sample. Foods.

[22] Um CY, Hodge RA, McCullough ML (2023). Change in diet quality and meal sources during the COVID-19 pandemic in a diverse subset of men and women in the cancer prevention study-3. Nutrients.

[23] Picchioni F, Goulao LF, Roberfroid D (2022). The impact of COVID-19 on diet quality, food security and nutrition in low and middle income countries: a systematic review of the evidence. Clin Nutr.

[24] Giorda CB, Forlani G, Manti R, Mazzotti A, De Cosmo S, Rossi MC (2018). Trend over time in hepatic fibrosis score in a cohort of type 2 diabetes patients. Diabetes Res Clin Pract.

[25] Marchisello S, Pino AD, Scicali R, Urbano F, Piro S, Purrello F (2019). Pathophysiological, molecular and therapeutic issues of nonalcoholic fatty liver disease: an overview. Int J Mol Sci.

[26] Chen P, Wu M, He Y, Jiang B, He ML (2023). Metabolic alterations upon SARS-CoV-2 infection and potential therapeutic targets against coronavirus infection. Signal Transduct Target Ther.

[27] Hamet P, Pausova Z, Attaoua R, Hishmih C, Haloui M, Shin J (2021). SARS–CoV-2 receptor ACE2 gene is associated with hypertension and severity of COVID 19: interaction with sex, obesity, and smoking. Am J Hypertens.

[28] Zhang V, Fisher M, Hou W, Zhang L, Duong TQ (2023). Incidence of new-onset hypertension post–COVID-19: comparison with influenza. Hypertension.

[29] Aghili SMM, Ebrahimpur M, Arjmand B, Shadman Z, Pejman Sani M, Qorbani M (2021). Obesity in COVID-19 era, implications for mechanisms, comorbidities, and prognosis: a review and meta-analysis. Int J Obes.

[30] American Diabetes Association Professional Practice Committee, ElSayed NA, Aleppo G, Bannuru RR, Bruemmer D, Collins BS, et al. 1. Improving Care and Promoting Health in Populations: *Standards of Care in Diabetes—2024*. Diabetes Care. 2024 1;47(Supplement_1):S11–9. 10.2337/dc24-S001 PMC10725798 38078573

[31] American Diabetes Association Professional Practice Committee, ElSayed NA, Aleppo G, Bannuru RR, Bruemmer D, Collins BS, et al. 12. Retinopathy, Neuropathy, and Foot Care: *Standards of Care in Diabetes—2024*. Diabetes Care. 2024;47(Supplement_1):S231–43. 10.2337/dc24-S012 PMC10725803 38078577

[32] Seid MA, Akalu Y, Gela YY, Belsti Y, Diress M, Fekadu SA (2021). Microvascular complications and its predictors among type 2 diabetes mellitus patients at Dessie town hospitals, Ethiopia. Diabetol Metab Syndr.

[33] Fawwad A, Mustafa N, Zafar AB, Khalid M. Incidence of microvascular complications of type 2 diabetes: A 12 year longitudinal study from Karachi-Pakistan. Pak J Med Sci. 2018;34(5). http://pjms.com.pk/index.php/pjms/article/view/15224 10.12669/pjms.345.15224 PMC6191805 30344550

[34] Malkani S, Keitz SA, Harlan DM (2016). Redesigning diabetes care: defining the role of endocrinologists among alternative providers. Curr Diab Rep.

[35] Jacob L, Rickwood S, Rathmann W, Kostev K (2021). Change in glucose-lowering medication regimens in individuals with type 2 diabetes mellitus during the COVID-19 pandemic in Germany. Diabetes Obes Metab.

[36] Chatterjee S (2020). SGLT-2 inhibitors for COVID-19—A miracle waiting to happen or just another beat around the bush?. Prim Care Diabetes.

[37] American Diabetes Association Professional Practice Committee, ElSayed NA, Aleppo G, Bannuru RR, Bruemmer D, Collins BS, et al. 9. Pharmacologic Approaches to Glycemic Treatment: *Standards of Care in Diabetes—2024*. Diabetes Care. 2024;47(Supplement_1):S158–78. 10.2337/dc24-S009 PMC10725810 38078590

[38] Mohan V, Saboo B, Khader J, Modi KD, Jindal S, Wangnoo SK (2022). Position of Sulfonylureas in the Current ERA: Review of National and International Guidelines. Clin Med Insights Endocrinol Diabetes.

[39] Sivaprasad S (2022). Epidemiological study on complications of diabetes and its treatment options. Asian J Pharm Technol..

[40] Baloch N, Gul A, Bhatti WS, Magsi I, Alahmadi YM, Almusalam AJ (2021). The frequency of diabetes related complications in patients with type 2 diabetes a case study of Tertiary Care Hospital. J Pharm Res Int..

[41] Babel RA, Dandekar MP (2021). A review on cellular and molecular mechanisms linked to the development of diabetes complications. Curr Diabetes Rev.

[42] Ong C (2022). Characteristic of chronic complications in type 2 diabetic patient based on Asian perspective. Curr Intern Med Res Pract Surabaya J.

[43] An J, Nichols GA, Qian L, Munis MA, Harrison TN, Li Z (2021). Prevalence and incidence of microvascular and macrovascular complications over 15 years among patients with incident type 2 diabetes. BMJ Open Diabetes Res Care.

[44] Galiero R, Caturano A, Vetrano E, Beccia D, Brin C, Alfano M (2023). Peripheral neuropathy in diabetes mellitus: pathogenetic mechanisms and diagnostic options. Int J Mol Sci.

